# A retrospective study on the analysis of influencing factors of neutropenia in endometrial cancer with adjuvant chemoradiotherapy

**DOI:** 10.1186/s13014-024-02469-8

**Published:** 2024-06-18

**Authors:** Mengsi Fan, Weiwei Zhang, Yuying Zhou, Mingzhuo Li, Dongyue Wang, Kexin Qiu, Mengzhen Li, Haoran Guo, Li Yan

**Affiliations:** 1 Department of Gynecology, Shandong Provincial Qianfoshan Hospital, Shandong Second Medical University, Key Laboratory of Laparoscopic Technology, the First Affiliated Hospital of Shandong First Medical University, Jinan, China; 2 School of Public Health, Shandong Second Medical University, Weifang, China; 3 Department of Gynecology, Tengzhou Maternal and Child Health Hospital, Tengzhou, China; 4 Center for Big Data Research in Health and Medicine, The First Affiliated Hospital of Shandong First Medical University & Shandong Provincial Qianfoshan Hospital, Jinan, China; 5 Shandong Data Open innovative Application Laboratory, Jinan, China; 6 School of Clinical Medicine, Shandong First Medical University, Jinan, China; 7 Department of Gynecology, Shandong Provincial Qianfoshan Hospital, Jinan, China

**Keywords:** Endometrial cancer, Chemoradiotherapy, Neutropenia, Neutrophil, Monocyte, Platinum, Docetaxel

## Abstract

**Objective:**

This retrospective study aimed to investigate the factors influencing the occurrence of neutropenia in patients with endometrial cancer (EC) following adjuvant chemoradiotherapy (CRT).

**Methods:**

Retrospective analysis of EC patients who underwent adjuvant CRT from January 2012 to June 2023 in the Department of Gynecology and Oncology of the First Affiliated Hospital of Shandong First Medical University. Neutropenia was defined as an Absolute Neutrophil Count (ANC) of peripheral blood neutrophils below 2 × 10^9^/L. Factors affecting neutropenia in EC patients treated with CRT using Generalized Estimating Equation (GEE), and Logistic regression was used to further analyze the effect of adding radiotherapy to different chemotherapy cycles on neutropenia, so that patients receive optimal adjuvant CRT while the risk of neutropenia is appropriately controlled.

**Results:**

A total of 144 patients met the inclusion criteria. They underwent 330 cycles of adjuvant chemotherapy, of whom 96 (66.7%) developed neutropenia, which occurred 140 times. The results of one-way GEE analysis showed that before CRT, White Blood Cell (WBC) (OR = 0.827; 95%CI, 0.701–0.976), ANC (OR = 0.749; 95%CI, 0.586–0.957), Absolute Monocyte Count (AMC) (OR = 0.047; 95%CI, 0.008–0.283), Blood Urea Nitrogen (BUN) (OR = 0.857; 95%CI, 0.741–0.991), platinum and docetaxel (platinum/docetaxel) dosing regimen (OR = 2.284; 95%CI, 1.130–4.618) were associated with neutropenia with adjuvant CRT for EC (*p* < 0.05), results of multifactorial GEE analysis showed that before adjuvant CRT ANC (OR = 0.552; 95%CI, 0.973–2.231), AMC (OR = 0.047; 95%CI, 0.004–0.052), platinum/docetaxel (OR = 2.437; 95%CI, 1.087–5.464) were an independent influence on neutropenia in adjuvant CRT for EC (*p* < 0.05). Multifactorial Logistic regression shows addition of radiotherapy to the first cycle of chemotherapy (OR = 4.413; 95%CI, 1.238–18.891) was an independent influence of neutropenia (*p* < 0.05).

**Conclusions:**

Patients with low pre-CRT ANC and AMC, platinum/docetaxel dosing regimens need to be closely monitored during each cycle of CRT. Also, the concurrent addition of radiotherapy should be avoided during the first cycle of chemotherapy.

## Introduction

Endometrial cancer is one of the three major malignant tumors of the female reproductive tract. Its incidence has been on the rise globally in recent years [1, 2]. In developed countries, the incidence of EC ranks first among gynecological malignancies and has become a serious problem endangering public health.

Although most patients are diagnosed at an early stage, high-risk, metastatic, and recurrent ECs remain important factors in patient survival. Currently, the treatment of endometrial cancer is mainly surgical, radiation and chemotherapy are commonly used as adjuvant treatments [3]. However, neutropenia is the most common hematologic toxicity during CRT, which can easily induce febrile neutropenia (FN), infectious toxic shock, and even death, impact on the clinical treatment and survival of tumor patients, but also increase the cost of medical care [4, 5]. Despite current progress in infection prevention, FN remains a common complication of chemotherapy in cancer patients [6].

The recent use of chemotherapy and radiotherapy techniques for various types of malignant tumors has led to a high incidence of neutropenia [7]. For patients with metastatic solid tumors such as metastatic breast cancer (MBC), colon/rectum cancer (MCRC), lung cancer (MLC), ovaries cancer (MOC), or prostate cancer (MPC) receiving standard myelosuppressive chemotherapy regimens, the incidence of FN ranges from 13 to 21%, with the highest occurrence in the first cycle (23–36%) [8]. In a retrospective study of stage III EC, the incidence of grade 4 neutropenia reached 73.8% in patients receiving CRT and 52.8% in those receiving chemotherapy alone [9]. According to a Japanese study, the incidence of FN in patients receiving concurrent CRT was 15% [10]. The prevalence of neutropenia ranges from 2 to 50% and is related to a variety of factors including patient-related risk factors, cancer type, chemotherapy, radiation therapy and genetic susceptibility [11].

As a result, many studies have attempted to identify risk factors for neutropenia and its consequences. The goal is to develop predictive models capable of identifying high-risk patients and guiding the application of more effective and cost-efficient granulocyte colony-stimulating factors (G-CSF) [12, 13]. For example, a retrospective analysis of 741 breast cancer patients who received adjuvant chemotherapy found that low ANC and Absolute Lymphocyte Count (ALC) were risk factors for FN [14]. Bartlett et al. noted that concurrent chemotherapy is one of the most important risk factors for radiation therapy-associated neutropenia [15]. A retrospective analysis by Mell et al. found that increases in pelvic bone marrow (PBM)-V (5), V (10), V (15), and V (20) were significantly associated with decreases in WBC and ANC nadirs in anal cancers treated with CRT [16]. A retrospective cohort study conducted at the Medical University of Graz demonstrated that among consecutive patients with testicular germ cell tumors (TGCT) treated with adjuvant cisplatin chemotherapy, higher age, and prior radiotherapy were associated with a higher risk of FN [17]. A European prospective observational study based on patients with non-Hodgkin’s lymphoma (NHL) presented a risk model for the development of FN in the first cycle of chemotherapy, clinical correlates significantly associated with FN in cycle 1 were: older age, planned dose increase of cyclophosphamide, history of prior chemotherapy, recent history of infection, low baseline albumin level [18]. Previous studies have also demonstrated the predictive value of low WBC, low Hemoglobin (Hb), or low Platelet (PLT) prior to the first chemotherapy cycle for neutropenia in subsequent cycles of chemotherapy [19, 20].

Although risk factors for the development of neutropenia following CRT in patients with different tumor types are currently assessed [21]. However, the risk factors for neutropenia in EC due to CRT are unclear. Therefore, with this study, aiming to explore the risk factors for neutropenia after CRT for EC, screening for more effective and cost-efficient prophylactic application of G-CSF in high-risk populations, more precise and rational prevention of neutropenia during CRT in patients with EC, thereby reducing patient hospitalization rates, lowering patient treatment costs, and increasing patient survival.

## Materials and methods

Approved by the Ethics Committee of the First Affiliated Hospital of Shandong First Medical University (Ethics No. YXLL-KY-2022(073)), retrospective analysis was performed on high-risk patients with EC who underwent surgery for EC in the First Affiliated Hospital of Shandong First Medical University from January 2012 to June 2023 and received adjuvant CRT after surgery in the department of gynecology and oncology in the hospital. Adjuvant CRT schedule: Sequential chemotherapy combined with concurrent chemoradiotherapy. Sequential chemotherapy is administered before or after concurrent chemoradiotherapy, depending on the patient’s postoperative recovery and general condition. High-risk EC were defined as fulfilling one of the following conditions: high-grade tumor (endometrioid grade 3 and nonendometrioid histologies), deep myometrial invasion (MI) (≥ 50%), or the presence of angiolymphatic invasion (LVSI).

Patients with histologically-proven EC were staged on the basis of final pathological findings according to the 2009 International Federation of Gynecology and Obstetrics (FIGO) classification. Inclusion criteria: (1) Histologically confirmed high-risk EC; (2) Age ≥ 18 years; (3) CRT treatment at our hospital; (4) No significant information missing. Exclusion Criteria: (1) Exclude those with unknown tumor stage, chemotherapeutic agents or cycles of chemotherapy; (2) Concomitant severe medical diseases; (3) Uncontrolled infections; (4) History of prior immunotherapy, history of stem cell transplantation. In this study, the subjects were strictly selected according to the inclusion and exclusion criteria, and the patients’ basic information, laboratory examination information, EC history information, adverse effects and supportation methods information were collected. Basic information includes: age, number of days of hospitalization, comorbidities (hypertension, diabetes, hyperlipidemia, coronary heart disease, cerebrovascular disease, etc.); Laboratory test information includes: before each CRT session Red Blood Cell (RBC), WBC, PLT, Hb, ALC, AMC, Serum Creatinine (SCR), BUN, before and after each CRT session ANC; EC history information includes: chemotherapy regimen, chemotherapy drugs, number of cycles of chemotherapy, radiotherapy dose, radiotherapy technique, and whether to add brachytherapy after external beam radiotherapy; Adverse effects and supportive care information comprise: infection, bone marrow suppression, G-CSF administration, antibiotics, and blood products. Index of outcome: neutropenia was defined as ANC less than 2 × 10^9^/L. Study groups: 2 groups were divided according to the occurrence of neutropenia or not. Study group: those who developed neutropenia after CRT; control group: those who did not develop neutropenia after CRT.

R 4.2.2 was used to complete the statistical analysis for this study. For measurement information: if the information follows a normal distribution, it is denoted by Mean (SD), and the t-test is used for comparison between two groups; If the information does not follow a normal distribution, it is denoted by M (Min, Max). Comparisons between groups were made using the independent samples Wilcoxon rank sum test. For count data: expressed as frequencies and percentages, comparisons between groups were made using the chi-square test or Fisher’s exact probability test. Factors influencing the development of neutropenia in patients undergoing CRT for EC were explored using GEE. A unifactorial GEE model was used to test each variable in the cohort, and those unifactorial variables that were statistically significant after testing were included in a multifactorial GEE model to screen for risk factors for neutropenia after CRT. Logistic regression models were used to analyze the effect of adding radiotherapy to different chemotherapy cycles on neutropenia. Univariate and multivariate analyses were performed using chi-square and Fisher exact tests. All statistical tests were two-sided, and a *p*-value of less than or equal to 0.05 was considered statistically significant.

## Results

A total of 144 patients with EC treated with CRT at our hospital from January 2012 to June 2023 met the inclusion criteria. The median age of the patients was 55.94 years with a standard deviation of 10.13 years (Table 1). Surgical FIGO stages I-IV were observed in 30 patients (20.83%) for stage I, 24 patients (16.67%) for stage II, 51 patients (35.42%) for stage III, and 26 patients (18.06%) for stage IV, due to the long-time span of the cases, the postoperative tumor stage records for 13 patients from earlier years are either lost or unclear. Histologic type was observed in 85 patients (59.03%) for endometrioid carcinoma, 24 patients (16.67%) for serous carcinoma, 10 patients (6.94%) for clear cell carcinoma, 13 patients (9.03%) for undifferentiated carcinoma, 3 patients (2.08%) for mixed cell carcinoma, 3 patients (2.08%) for mucinous carcinoma, and 6 patients (4.17%) for carcinosarcoma. The majority of patients (56.94%) were treated with a platinum/paclitaxel dosing regimen. Platinum agents primarily include carboplatin and cisplatin, while taxanes mainly consist of paclitaxel albumin-bound, paclitaxel liposome, and conventional paclitaxel.

**Table 1:** Primary study measures and baseline characteristics of patients

Variables	Mean (SD)/ Median (Min, Max)/ (*N*%)
Age, years	55.94 (10.13)
Hb, g/L	120 (66,152)
PLT, ×10^9^ /L	239.39 (78.17)
WBC, ×10^9^ /L	5.26 (2.43,16.14)
ALC, ×10^9^ /L	1.46 (0.54)
RBC, ×10^9^ /L	4.03 (2.3,5.02)
AMC, ×10^9^ /L	0.38 (0.11,1.92)
ANC, ×10^9^ /L	3.33 (0.56,13.99)
SCR, umol/L	56 (35.5,147)
BUN, mmol/L	4.4 (1.5,20.23)
Tumor stage	
I	30 (20.83%)
II	24 (16.67%)
III	51 (35.42%)
IV	26 (18.06%)
Unknown^a^	13 (9.03%)
Histologic type	
Endometrioid carcinoma	85 (59.03%)
Serous carcinoma	24 (16.67%)
Clear cell carcinoma	10 (6.94%)
Undifferentiated carcinoma	13 (9.03%)
Mixed cell carcinoma	3 (2.08%)
Mucinous carcinoma	3 (2.08%)
Carcinosarcoma	6 (4.17%)
Chemotherapeutic regimen	
Platinum and paclitaxel	82 (56.94%)
Platinum and docetaxel	43 (29.86%)
Platinum and tapiocin	5 (3.47%)
Others	14 (9.72%)

Note: ^a^ is the absence of pathological type results

Abbreviations: Hb = Hemoglobin; PLT = Platelet; WBC = White Blood Cell; ALC = Absolute Lymphocyte Count; RBC = Red Blood Cell; AMC = Absolute Monocyte Count; ANC = Absolute Neutrophil Count; SCR = Serum Creatinine; BUN = Blood Urea Nitrogen

For postoperative radiotherapy in EC, our hospital primarily utilizes two radiation therapy techniques: Intensity Modulation Radiation Therapy (IMRT) and brachytherapy. The total doses are 45–50 Gy/1.8–2.0 Gy/25–28 F, 21 Gy/7Gy/3 W or 30 Gy/6Gy/5 W or 30 Gy/ 5 Gy/6 W, respectively. A total of 141 patients (97.92%) received IMRT radiation therapy, among whom 26 patients (18.44%) underwent brachytherapy after IMRT, and 3 patients (2.08%) received brachytherapy alone. Due to the wide time span of case collection, records for 17 patients regarding radiation techniques were unclear, and it was uncertain whether brachytherapy was used after IMRT. Four patients developed herpes zoster, six patients experienced enteritis and received symptomatic treatment. Ten patients had urinary tract infections and were treated with antibiotics, with one patient developing bacteremia. Three patients underwent PLT transfusions. Sixty-nine patients experienced bone marrow suppression and were treated with G-CSF. One patient had a low Hb level of 64.0 g/L and received symptomatic treatment with recombinant human erythropoietin injections.

Univariate GEE analysis showed that WBC, ANC, AMC, BUN, and platinum/docetaxel dosing regimen before CRT were influential factors in the occurrence of neutropenia (*P* < 0.05). (Table 2).

**Table 2:** One-way GEE analysis

Variables	OR (95%CI)	*P*
Age, years	0.968 (0.935–1.003)	0.070
Hb, g/L	1.008 (0.992–1.024)	0.322
WBC, ×10^9^ /L	0.827 (0.701–0.976)	0.024
ANC, ×10^9^ /L	0.749 (0.586–0.957)	0.021
AMC, ×10^9^ /L	0.047 (0.008–0.283)	0.001
PLT, ×10^9^ /L	0.998 (0.994–1.001)	0.218
RBC, ×10^9^ /L	0.982 (0.659–1.463)	0.930
ALC, ×10^9^ /L	1.008 (0.685–1.483)	0.966
SCR, umol/L	0.991 (0.976–1.007)	0.281
BUN, mmol/L	0.857 (0.741–0.991)	0.037
Chemotherapeutic regimen		
Platinum and paclitaxel	1.000	
Platinum and docetaxel	2.284 (1.130–4.618)	0.021
Platinum and tapiocin	0.804 (0.235–2.747)	0.727
Others	0.811 (0.371–1.770)	0.598

Abbreviations: Hb = Hemoglobin; PLT = Platelet; WBC = White Blood Cell; ALC = Absolute Lymphocyte Count; RBC = Red Blood Cell; AMC = Absolute Monocyte Count; ANC = Absolute Neutrophil Count; SCR = Serum Creatinine; BUN = Blood Urea Nitrogen

Multifactorial GEE analysis showed that ANC, AMC, and platinum/docetaxel dosing regimen before CRT were independent influences on the occurrence of neutropenia (*P* < 0.05) (Table 3).

**Table 3:** Multifactor GEE analysis

Variables	OR (95%CI)	*P*
WBC, ×10^9^ /L	1.446 (0.937–2.231)	0.096
ANC, ×10^9^ /L	0.552 (0.318–0.958)	0.035
AMC, ×10^9^ /L	0.047 (0.004–0.502)	0.011
BUN, mmol/L	0.879 (0.729–1.060)	0.178
Chemotherapeutic regimen		
Platinum and paclitaxel	1.000	
Platinum and docetaxel	2.437 (1.087–5.464)	0.031
Platinum and tapiocin	0.817 (0.265–2.524)	0.726
Others	0.969 (0.410–2.288)	0.943

Abbreviations: WBC = White Blood Cell; AMC = Absolute Monocyte Count; ANC = Absolute Neutrophil Count; BUN = Blood Urea Nitrogen

**Fig. 1:**
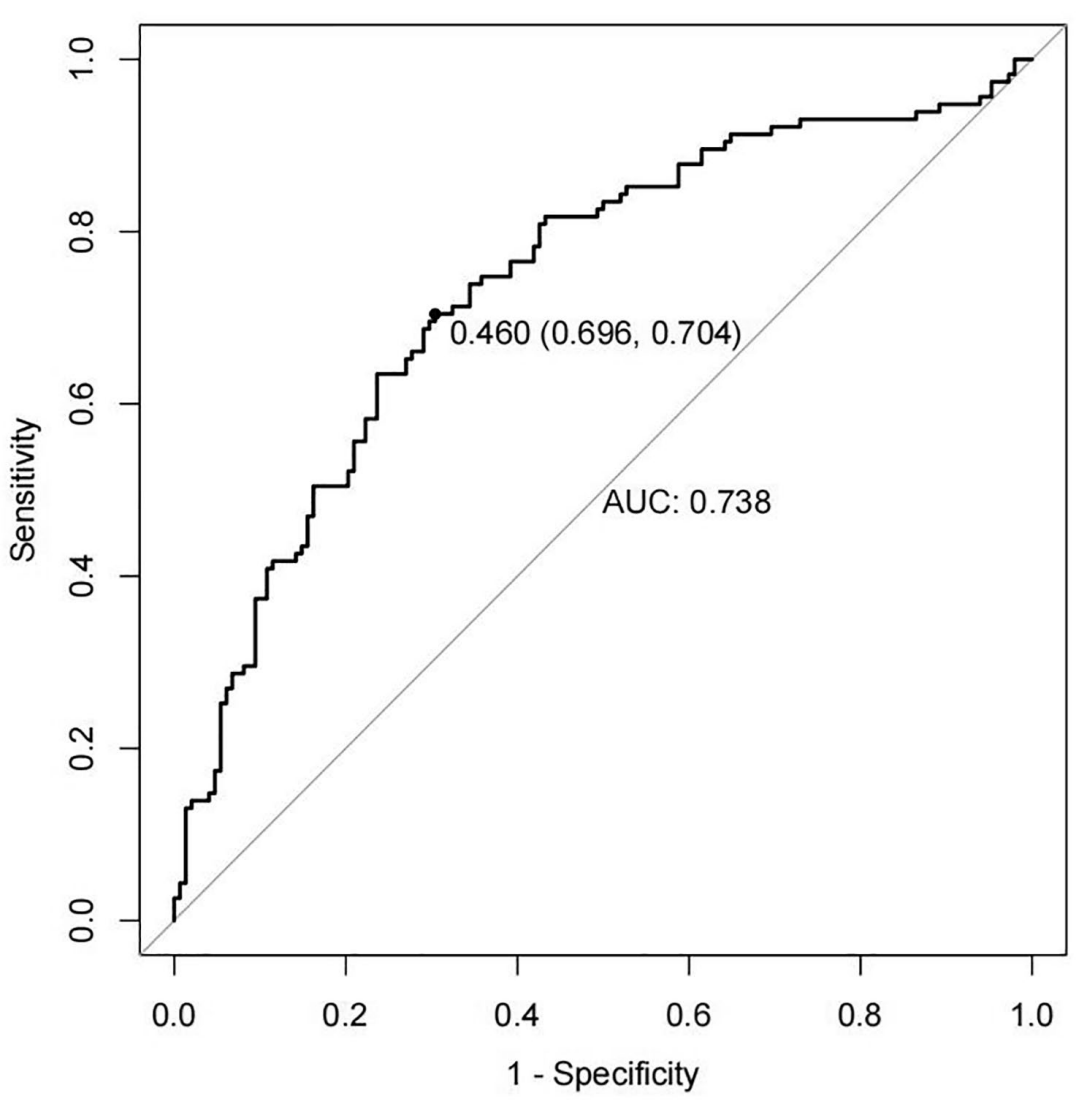
Receiver Operating Characteristic

Receiver Operating Characteristic (ROC) analysis was employed to evaluate the predictive capability of each laboratory index before each CRT session regarding neutropenia after CRT (Fig. 1). The model demonstrated good discriminative performance, with an ROC area of 0.738. The sensitivity for neutropenia was 70.4%, and the specificity was 69.6%.

Neutropenia was observed in 69 individuals during the first cycle, 32 individuals during the second cycle, 16 individuals during the third cycle, and 10 individuals during the fourth cycle, with the majority of cases occurring in the first cycle. The inclusion of radiotherapy in the first cycle of chemotherapy had an independent impact on the occurrence of neutropenia (*P* < 0.05) (Table 4).

**Table 4:** Addition of radiotherapy to different chemotherapy cycles

Chemotherapy cycle	OR (95%CI)	*P*
First cycle	4.413 (1.238–18.891)	0.034
Second cycle	6.740 (1.043-132.497)	0.088
Third cycle	2.400 (0.513–17.143)	0.303
Fourth cycle	4.091 (0.611–80.905)	0.211

## Discussion

### Principal findings

Although numerous studies have evaluated the factors influencing neutropenia and/or FN after CRT in patients with different tumor types, no studies have been conducted on the factors influencing neutropenia after CRT in patients with EC. Additionally, there are several limitations to previous studies, including some studies that only explore factors influencing neutropenia on the first cycle of chemotherapy, and most statistical analyses are primarily based on classical statistical methods. However, EC patients typically undergo multiple cycles of chemotherapy during the course of CRT, and traditional statistical models are unable to cope with such longitudinal information of repeated measures.

Our study used the GEE model for the first time to explore the factors influencing the occurrence of neutropenia after adjuvant CRT in EC patients. The GEE model is proposed by Liang & Zeger (1986) a method for analyzing longitudinal data based on generalized linear models [22]. We reached two main conclusions: (1) Pre-CRT ANC, AMC, and platinum/docetaxel dosing regimen are independent influences on the occurrence of neutropenia. (2) The addition of radiotherapy to the first cycle of chemotherapy is an independent influence on the development of neutropenia.

### Results in the context of what is known & clinical implications & Research implications

Most studies have reported the incidence of postoperative CRT-induced neutropenia and/or FN in patients with EC. Milgrom et al. retrospectively analyzed patients with stage III EC who received postoperative adjuvant CRT. Of the 40 eligible patients, 4 (10%) were neutropenic [23]. Wen et al. treated 31 patients with high-risk or advanced EC using paclitaxel plus carboplatin in combination with CRT, and 45.2% of the patients developed grade 3/4 neutropenia [24]. A study of high-risk ECs noted that for patients who have received radiotherapy alone and combined CRT, grade 3 neutropenia occurred in 9.8% of patients in both groups [25]. However, limited to reports on the incidence of neutropenia in EC CRT, there are no clear reports on the factors influencing the occurrence of neutropenia in EC patients after CRT.

Myelosuppression can be induced by cytotoxic chemotherapeutic agents as well as by radiation therapy in addition to chemotherapy, usually affecting all blood cell lines, including ANC, APL, Hb and ALC. Many studies have found that abnormal laboratory test values are an important influence on neutropenia after chemotherapy [26]. Previous studies have shown that pre-chemotherapy Total Bilirubin (TBil) ≥ 25µmol/L, ALC < 0.7 × 10^9^/L are independent risk factors for the development of neutropenia after chemotherapy [27]. Corbeau et al. found that baseline low PLT, low WBC, and low ANC were risk factors for neutropenia after chemotherapy, and that the ANC to ALC ratio was an independent predictor of FN [28]. A prospective study of Hodgkin’s lymphoma noted a possible increased risk of FN in patients with an AMC below 25 × 10^6^/L on day 8 after chemotherapy [29]. In another similar study, Shimanuki et al. investigated the use of pretreatment hematologic laboratory parameters to construct a predictive model for chemotherapy-induced FN in patients with squamous cell carcinoma of the head and neck, the model shows, that AMC values less than 370/mm^3^ are good predictors of chemotherapy-induced FN [30]. In addition, a baseline AMC of less than 150/µL has been identified as a poor prognostic variable in patients with chemotherapy-induced FN [31]. Oguz et al.reported that in solid childhood tumors, an ALC ≤ 0.7 × 10^9^/L on day 5 and an AMC ≤ 0.15 × 10^9^/L on day 7 were independent risk factors for FN [32]. Our research has found that pre-CRT ANC, AMC is an independent influence on the occurrence of neutropenia in patients with EC, which is in line with the findings of previous studies. Unlike previous studies, which mostly focused on the effects of chemotherapy leading to the development of neutropenia, our study added synchronized radiation therapy.

Many studies have found that certain chemotherapy drugs and regimens promote myelosuppression more than others [33, 34]. The current National Comprehensive Cancer Network (NCCN) guidelines state that carboplatin in combination with paclitaxel is the preferred chemotherapy regimen for the treatment of advanced, metastatic or recurrent EC [35], other commonly used regimens or drugs include docetaxel in combination with carboplatin, doxorubicin in combination with cisplatin, carboplatin in combination with a paclitaxel regimen plus bevacizumab, liposomal doxorubicin, albumin-bound paclitaxel, topotecan, etc., and in patients with contraindications to paclitaxel, docetaxel in combination with carboplatin may be considered. In our study, building from previous research, patients were treated with standard chemotherapy regimens along with the addition of radiation therapy, we set patients on platinum and paclitaxel-based chemotherapy regimens as the control group, receiving a platinum/docetaxel chemotherapy regimen compared to platinum/paclitaxel is a risk factor for neutropenia in patients treated with CRT for EC. Previous studies have also shown that anthracyclines (e.g., doxorubicin), taxanes (e.g., docetaxel), alkylators (e.g., cyclophosphamide), and topoisomerase inhibitors (e.g., etoposide) as well as gemcitabine and vinorelbine are particularly myelosuppressive [13]. It has also been illustrated that docetaxel has a relatively high incidence of FN compared to other cytotoxic anticancer drugs [36]. Our study is consistent with previous findings.

Currently, radiation therapy in addition to chemotherapy is known to increase the incidence of myelosuppression. However, determining the optimal timing to introduce radiation therapy during chemotherapy to reduce the occurrence of myelosuppression is an issue that requires further investigation. Previous studies have found that the risk of neutropenia in most chemotherapy patients is greatest in the initial chemotherapy cycle, with a reduced incidence of neutropenia in subsequent treatment cycles [37]. A prospective multinational study of the incidence of FN in patients with solid tumors or Hodgkin’s/non-Hodgkin’s lymphoma at intermediate risk for FN receiving chemotherapy found that the incidence of FN is higher in cycle 1 and decreases in subsequent cycles, with the incidence of FN being 5% in cycle 1, 3% in cycles 2–3 and 1% in cycles 4–6 [38]. A prospective study of a multivariate analysis of FN in NHL patients concluded that myelosuppression, especially FN, is a severe dose-limiting toxicity that frequently occurs during the first cycle of chemotherapy. In a study reviewing the medical records of 1,355 patients with moderate NHL who received cyclophosphamide, adriamycin, vincristine, and prednisone or similar chemotherapy, more than half of the first hospitalizations for FN occurred in cycle 1 or 2 [39]. In our study, neutropenia occurred in 69 people in the first cycle, 32 people in the second cycle, 16 people in the third cycle, 10 people in the fourth cycle, most of the patients experienced neutropenia in the first cycle, consistent with previous studies. At the same time, this indirectly confirms the results of our study, the addition of radiotherapy to the first cycle of chemotherapy is an independent risk factor that exacerbates the development of neutropenia. Therefore, the concurrent addition of radiotherapy should be avoided during the first cycle of chemotherapy. The same study on breast cancer also suggested that FN is associated with the concurrent use of breast chemotherapy and radiation therapy, and recommended avoiding concurrent use of chemotherapy and radiation therapy for breast cancer [40].

### Strengths and limitations

The novelty of this study compared to previous studies is that it is the first to examine the risk factors for neutropenia due to CRT for EC. In addition, since patient outcomes after each CRT session, as well as laboratory test indices and medication regimens before each CRT session, involve multiple measurements constituting longitudinal data, the correlation between variables cannot be addressed by traditional statistical methods. Therefore, we employed a GEE model specifically designed for longitudinal cohorts to analyze repeated measurements within the same patient. The GEE model well solves the correlation problem of longitudinal data utilizing each measurement in longitudinal data greatly reduces the loss of information and allows for robust parameter estimates. The limitations of this study are that although chemotherapy for women with EC averages at six cycles, we were more likely to only be able to collect outcome data for the first four cycles of chemotherapy due to the fact that some of our patients did not have regular treatment. In addition to this, because this study is a single-institution retrospective study, the data in this study came from a single healthcare organization, which is a single-center study, and there may be some limitations in extrapolating the findings of the study, thus multi-institutional studies and other external cohorts are needed to validate it in the future.

## Conclusions

We have identified several pre-treatment patient and treatment-related factors that predict the occurrence of neutropenia events during CRT for EC. Low ANC and AMC prior to CRT, platinum/docetaxel dosing regimen, and concurrent radiotherapy during the first cycle of chemotherapy were associated with neutropenia in EC.

## Data Availability

The datasets used and/or analyzed during the present study are available from the corresponding author on reasonable request.

## Abbreviations

EC: Endometrial cancer
CRT: Chemoradiotherapy
ANC: Absolute Neutrophil Count
GEE: Generalized Estimating Equation
WBC: White Blood Cell
AMC: Absolute Monocyte Count
BUN: Blood Urea Nitrogen
FN: Febrile neutropenia
G-CSF: Granulocyte colony-stimulating factors
ALC: Absolute Lymphocyte Count
Hb: Hemoglobin
PLT: Platelet
RBC: Red Blood Cell
SCR: Serum Creatinine
PBM: Pelvic bone marrow
TGCT: Testicular germ cell tumors
NHL: non-Hodgkin’s lymphoma
MI: Myometrial invasion
LVSI: Angiolymphatic invasion
FIGO: International Federation of Gynecology and Obstetrics
TBil: Total Bilirubin
NCCN: National Comprehensive Cancer Network
